# The Current Status and Prospects of the Application of Omics Technology in the Study of *Ulmus*

**DOI:** 10.3390/ijms252312592

**Published:** 2024-11-23

**Authors:** Shijie Wang, Lihui Zuo, Yichao Liu, Lianxiang Long, Min Jiang, Mengjuan Han, Jinmao Wang, Minsheng Yang

**Affiliations:** 1 Institute of Forest Biotechnology, College of Forestry, Hebei Agricultural University, Baoding 071000, China; 2 Hebei Key Laboratory for Tree Genetic Resources and Forest Protection, Baoding 071000, China; 3 School of Landscape and Ecological Engineering, Hebei University of Engineering, Handan 056038, China; 4 Institute of Landscaping, Hebei Academic of Forestry and Grassland, Shijiazhuang 050061, China

**Keywords:** abiotic stress, biotic stress, growth and development, omics technologies, systematic evolution, *Ulmus*

## Abstract

Elm (*Ulmus*) species are important components of forest resources with significant ecological and economic value. As tall hardwood trees that are drought-resistant, poor-soil-tolerant, and highly adaptable, *Ulmus* species are an excellent choice for ecologically protected forests and urban landscaping. Additionally, the bioactive substances identified in the fruits, leaves, bark, and roots of *Ulmus* have potential applications in the food and medical fields and as raw materials in industrial and cosmetic applications. However, the survival of *Ulmus* species in the natural environment has been threatened by recurrent outbreaks of Dutch elm disease, which have led to the death of large numbers of *Ulmus* trees. In addition, severe damage to the natural habitats of some *Ulmus* species is driving their populations to extinction. Omics technology has become an important tool for the collection, protection, and biological characteristic analysis of *Ulmus* species and their resources due to its recent advances. This article summarizes the current research and application status of omics technology in *Ulmus*. The remaining problems are noted, and future research directions are proposed. Our review is aimed at providing a reference for resource conservation of *Ulmus* and for scientific research into this genus.

## 1. Introduction

Elm (*Ulmus*) is an ancient tree genus that can be traced back to the Tertiary period and contains abundant germplasm resources [1]. Over 40 species have been recognized worldwide, with the majority of them distributed in northern Asia, North America, and Europe. Most *Ulmus* species are light-loving, with highly developed root systems that confer high drought resistance. Although the majority of *Ulmus* species are not resistant to high water and moisture levels, they do not have strict soil requirements [2]. These biological characteristics enable *Ulmus* to play a crucial role in maintaining ecological balance and protecting biodiversity [3,4,5]. Most *Ulmus* species are valued as a high-end material for their hard, fine-textured wood and in garden landscapes for their beautiful crown shape [6,7]. In addition, *Ulmus* bark, leaves, fruits, and roots contain polysaccharides, polyphenols, and other bioactive substances of value in drugs and foods [8,9]. *Ulmus* species are therefore not only of major ecological value but also of high economic importance, with considerable potential for further development and use. Nonetheless, research into *Ulmus* applications lags behind that of other tree species.

The rapid development of analytical technologies such as high-throughput sequencing, mass spectrometry, and nuclear magnetic resonance spectroscopy has led to major improvements in omics research while substantially reducing its cost [10,11]. The field of biological breeding is thus increasingly relying on omics data, including the impressive progress made in whole-genome sequencing. As of 2021, whole-genome sequencing has been completed for as many as 218 tree species worldwide [12]. This has greatly promoted the in-depth development and efficient use of forest resources and also signifies that forest research has entered the post-genomics era. However, despite the importance of *Ulmus* species as a forest resource, whole-genome sequencing of *Ulmus* has not yet been reported, greatly limiting the development and use of *Ulmus* resources.

Nevertheless, chloroplast genome sequencing, simplified genome sequencing, transcriptome sequencing, and metabolomics have been conducted for *Ulmus* in omics studies covering but not limited to phylogenetics, genetic diversity, growth and development, leaf color changes, and biotic and abiotic stress responses. This review examines recent achievements in *Ulmus* omics studies, with the aim of providing a better understanding of the biological characteristics of *Ulmus*. It also offers suggestions and references for the protection of *Ulmus*, the use of its genetic resources, and research into its functional genes.

## 2. *Ulmus* Plant Genomics Research

Plant cells contain both nuclear and organelle genomes, with the former accounting for 80–90% of the total DNA content, while the remaining DNA is mainly dispersed in plastids and mitochondria [13]. The smaller size and simpler structure of organelle genomes make them easier to sequence and assemble, such that a large number of plant organelle genomes have been published. Nuclear genomes, by contrast, are highly complex, have numerous repetitive sequences, and are difficult to assemble, resulting in high sequencing costs and, thus, the need for sufficient funding and support from large research institutions. For *Ulmus*, many organelle genomes have been sequenced, resulting in substantial progress in systematic evolutionary research focused on this genus. By contrast, published sequences of the nuclear genome of *Ulmus* are mostly lacking.

### 2.1. Progress in the Study of the Chloroplast Genomes of Ulmus

For the majority of plants, chloroplasts are semi-autonomous organelles with independent genetic material passed on via matrilineal inheritance, such that the chloroplast genome structure is highly conserved [14,15]. For tree species lacking nuclear genomic data, comparative analyses of chloroplast genome sequences among different species can provide valuable reference information for species identification, genus classification, and phylogenetic studies.

The complete sequence of the chloroplast genome of *Ulmus* was first reported in 2017 [16]. Since then, research on the chloroplast genome of *Ulmus* has progressed rapidly. According to our search of the NCBI database and the relevant literature, as of June 2024, the chloroplast genomes of 34 *Ulmus* species (Table 1), cultivars, and varieties have been published [16,17,18,19]. Based on those studies, the chloroplast genomes of *Ulmus* have a typical tetrad structure, including one large single copy (LSC), one small single copy (SSC), and two equally sized inverted repeat (IR) regions. The chloroplast genome size of *Ulmus* ranges from 158 to 160 kb, with the lengths of the LSC, SSC, and IRs in the range of 87–89 kb, 18–20 kb, and 26–27 kb, respectively, and a GC content (Guanine–Cytosine ratio) of 35–37%. The chloroplast genome of *Ulmus* is relatively conserved, with high similarities in gene quantity and sequence and few differences among species [17,19].

Most research on the chloroplast genomes of *Ulmus* has focused on phylogenetics. Such studies have shown that species in the Ulmaceae family have close phylogenetic relationships with those in the Moraceae and Cannabaceae families [16,20]. However, there are differences between the phylogenetic results obtained for *Ulmus* and traditional taxonomic views [2]. Overall, *Ulmus* can be divided into two branches. The first branch includes sections *Ulmus* and *Microptelea*, and the second sections *Trichoptelea*, *Chaetoptelea*, and *Blepharocarpus*, with a nesting relationship between the different sections to some extent [17,19]. According to phylogenetics, *U. lanceifolia*, an evergreen, should be grouped separately, not under section *Ulmus*, consistent with the research of Wiegrefe et al. [19,21]. Several other findings do not align with traditional taxonomic views. For instance, traditional taxonomy considers *U. davidiana* var. *japonica* as a variety of *U. davidiana*, but evolutionary results based on the chloroplast genome show that these two taxa do not cluster on a single branch [16]. This is also the case for *U. pumila* and *U. pumila* cv. ‘Zhonghuajinye’ [18].

Microsatellite (or simple sequence repeat, SSR) marker analysis, codon usage bias analysis, gene loss analysis, and positive selection pressure analysis have also been used to study the chloroplast genome of *Ulmus*. The number of SSR loci was shown to range from 110 to 130, with most distributed in the LSC and SSC regions and the fewest in the IRs, where >80% of the SSR loci belong to single-nucleotide loci. These SSR loci can serve as molecular markers for phylogenetic and population genetics studies of *Ulmus* [17,18]. Furthermore, distinct codon preference in the protein-coding genes of the chloroplast genomes of *Ulmus* species has also been demonstrated, including for *U. pumila*, *U. laciniata*, *U. davidiana*, *U. davidiana* var. *japonica*, and *U. macrocarpa*, with a higher relative frequency of usage for TTT, AAT, AAA, ATT, and TTC [16]. Positive selection pressure analysis suggests that some genes in the *Ulmus* chloroplast genome are subject to environmental selection, such as *atpF*, *rps15*, and *rbcL*, and have played crucial roles in the evolution of *Ulmus* [17,18]. Aziz et al. examined the chloroplast genomes in American and Asian *Ulmus* species and found that *petB*, *petD*, *psbL*, *rps16*, and *trnK* are present only in American *Ulmus,* whereas *trnH* is present in most Asian *Ulmus* but not in American *Ulmus* [20].

### 2.2. Progress in the Study of the Nuclear Genome of Ulmus

In 2006, the publication of the *Populus trichocarpa* genome marked the entry of woody plants into the genomics era [22]. With the subsequent release of the T2T genome and super pan genome of *Populus*, the study of woody plant genomes has further progressed [23,24,25,26]. By contrast, few advancements have been made in *Ulmus* nuclear genome research, and no genome of any *Ulmus* species has been reported. Nonetheless, research on the genomes of multiple *Ulmus* species is being conducted, including *Ulmus glabra* (PRJEB75992), *U. americana* (PRJNA390847), *U. minor*, and *U. pumila* [27,28].

Studies have shown that the chromosome karyotype of *Ulmus* follows a chromosome base of X = 14. Most *Ulmus* plants are diploid, with a chromosome number of 28, and no aneuploid variation has been observed [29,30,31,32]. Under natural conditions, polyploidization is rare in *Ulmus*, with *U. americana* being a special case as it includes both diploid individuals with 28 chromosomes and tetraploid individuals with 56 chromosomes [33,34]. Zhang et al. successfully induced tetraploidy in *U. pumila* using colchicine; light energy utilization efficiency and net photosynthetic rate were significantly higher in the tetraploid plants than in the diploid plants [35]. However, variations in the chromosome number of *Ulmus* plants are not limited to diploidy and tetraploidy; triploids have been observed in some species, including *U. americana*, *U. glabra*, *U. pumila*, and other species [34,36,37]. These findings suggest that polyploid breeding techniques can be applied in the genetic improvement of *Ulmus*.

Genome size is an important factor in genome sequencing, population diversity analysis, and studies of interspecific parentage relationships [38]. Using flow cytometry, Whittemore et al. found a wide variation in the genome size of the 33 analyzed *Ulmus* species, ranging from *U. wallichiana*, with has a haploid genome of 2.037 Gb, to *U. villosa*, with a haploid genome of 1.064 Gb (Table 2). The results of that study also provided strong molecular evidence for the subgenus classification of *Ulmus* species. For example, they showed that the genome size of various *Ulmus* plants within the same subgenus was basically the same; the average genome size of 23 species in subgenus *Ulmus* was 1.897 Gb, and that of nine species in subgenus *Oreoptelea* was 1.520 Gb, representing a difference of approximately 30%. In addition, because of the large genomic differences between *U. villosa* and other *Ulmus* species, *U. villosa* was proposed as a third subgenus [39]. However, we found that the experimental results and sequencing assembly results for certain *Ulmus* species reported by Whittemore et al. were inconsistent with the literature. In the NCBI database, the genome of *U. americana* has been updated in three versions, with sizes ranging from 0.865 to 1.3 Gb, whereas according to Whittemore et al., the haploid gene size of *U. americana* ranges from 1.469 to 1.607 Gb. A similar problem was identified for *U. minor*. According to the literature, the size of its preliminary genome is 1.09 Gb, whereas Whittemore et al. reported a size range of the haploid genome of 1.821–2.007 Gb [28]. Given that the final genome sequences of *U. americana* and *U. minor* have not yet been published, the authenticity of these differences has yet to be verified.

DNA molecular markers are a new generation of genetic markers developed based on nucleotide sequence differences, reflecting differences at the DNA level. They are an important tool in studies of the genetic diversity of species at the genome level [40,41]. In *Ulmus*, DNA molecular marker technology, including molecular marker techniques such as amplified fragment length polymorphism (AFLP), randomly amplified polymorphic DNA (RAPD), restriction fragment length polymorphism (RFLP), inter simple sequence repeat (ISSR), sequence-related amplified polymorphism (SRAP), and SSR, has been extensively applied in genetic diversity studies, phylogenetic analysis, species identification, genetic relationship determinations, pathogen identification, trait association analysis, linkage mapping of disease resistance genes, environmental adaptability studies, and to assess endangered species protection measures (Table 3). In addition, with the aid of the new generation of simplified genome sequencing technology, a large number of single-nucleotide polymorphism (SNP) and small-fragment insertion/deletion variation (Indel) markers can be developed, which will facilitate studies of *Ulmus* species lacking a reference genome. Whittemore et al. used restriction site-associated DNA sequencing technology to construct simplified genomes for multiple *Ulmus* species. Using SNP and Indel markers, they analyzed the phylogenetic and taxonomic relationships of more than 30 *Ulmus* species, from which they inferred the migration of *Ulmus* species across continents by combining morphological variations and hybridization relationships among species. The authors suggested dividing *Ulmus* into three subgenera and six sections and that *U. villosa* should be classified as subgenus *Indoptelea*, consistent with their previous research findings [39,42]. Lyu et al. used specific locus amplified fragment sequencing technology to analyze the SNP loci of 107 *U. parvifolia* individuals in seven populations. Moderate genetic diversity and little genetic differentiation were demonstrated among populations. Furthermore, association analysis of phenotypic traits and SNP loci identified a number of genes related to environmental adaptation. These studies have deepened our understanding of the genetic diversity and environmental adaptation of *U. parvifolia* and other *Ulmus* species [43].

## 3. *Ulmus* Transcriptomics

Transcriptome analysis can reveal the expression patterns of genes. By comparing gene expression in different tissues or physiological states, the molecular mechanism of specific biological processes can be explored. Transcriptome technology has been used widely in *Ulmus* studies, including research into its molecular markers, growth and development, leaf color change, stress response, abiotic stress response, and adaptive evolution (Table 4). However, the focus of those studies was only on a few species, mostly *U. pumila*, *U. pumila* cv. ‘Zhonghuajinye’, *U. americana*, *U. minor*, and *U. wallichiana*. Moreover, most transcriptomic studies on *Ulmus* species involved non-reference transcriptomes. This approach involves reconstructing gene sequences and functionalities mainly through reads assembly and subsequent alignment with databases, such as the non-redundant protein database (NR) and universal protein database (UniProt). As a result, there is room for improvement in the accuracy of gene function annotation and quantitative analysis in these studies.

### 3.1. Growth and Development

The wood of *Ulmus* is known for its wear- and corrosion-resistant properties, which accounts for its use in landscape decoration, furniture manufacturing, and shipbuilding. In addition, the leaves and fruits of *Ulmus* are of high nutritional value and potential medicinal interest. Studying *Ulmus* growth and development through transcriptomic methods will provide a deeper understanding of their unique developmental mechanisms and accelerate the creation of new cultivars. Comparisons of branch growth from different *Ulmus* varieties and at different periods have shown that phenylpropanoid biosynthesis and the lignin metabolic pathway play significant roles in branch thickening [6]. From February to March, the cambium of *Ulmus* branches resumes growth, accompanied by the expression of genes such as *CDKB*, *CYCB*, *WOX4*, and *ARF5*. From May to June, carbon allocation in *Ulmus* shifts from sugar synthesis to cellulose and lignin synthesis. This period is marked by the up-regulation of genes related to cellulose, xylan, and lignin biosynthesis [63]. Research on *Ulmus* fruit shows that genes related to the biosynthesis of unsaturated fatty acids and jasmonic acid are involved in the development of elm fruit, whereas genes related to starch and sucrose synthesis, which enhance nutrient accumulation, are expressed during the late stage of fruit ripening [62,66]. In addition, the expression patterns of a series of key genes and metabolic pathways in the biological processes of tissue aging in elm trees have been extensively studied. Genes such as *SWEET1*, *SCPL*, *SAG29*, *ERF019*, and *GALT6* are differentially expressed during leaf senescence, which may be closely related to its molecular regulation [77]. Research on the aging of *Ulmus* seeds has shown the differential expression of genes related to endoplasmic reticulum protein processing, plant hormone signaling transduction, the MAPK signaling pathway, and oxidative phosphorylation. Seed aging has also been linked to microRNAs (miRNAs) [69]. In a study in which the cellular microstructure and transcriptome were used to investigate the potential mechanism of growth inhibition in *U. pumila* cv. ‘Zhonghuajinye’, abnormalities in chloroplasts structure were detected, including the grana lamella stacking failures and fewer thylakoid grana slice layers. In addition, decreases in light energy absorption, conversion, and transport, carbon dioxide fixation, lipopolysaccharide biosynthesis, auxin synthesis, and protein transport in *U. pumila* cv. ‘Zhonghuajinye’ compared to *U. pumila* were determined. Conversely, genes related to respiration and starch consumption were found to be more highly expressed in *U. pumila* cv. ‘Zhonghuajinye’. This expression pattern may serve to inhibit the growth of *U. pumila* cv. ‘Zhonghuajinye’ [73].

### 3.2. Leaf Color Changes

*Ulmus* trees have an elegant shape and a graceful presence, which accounts for their high ornamental and cultural value [86]. *U. pumila* cv. ‘Zhonghuajinye’ is characterized by its golden yellow foliage, fine and dense branches and leaves, and suitability as both a tall tree and shrub [87]. However, under certain environmental conditions, the leaf color of *U. pumila* cv. ‘Zhonghuajinye’ undergoes significant changes. Specifically, when the light intensity decreases, the golden yellow leaves gradually revert to green; under high temperature or high light intensity, the leaves turn white [74,88,89,90]. This change reflects the dynamic instability of leaf color in *U. pumila* cv. ‘Zhonghuajinye’, a property that has been exploited in studies aimed at elucidating the coloration mechanism of its leaves. For example, the expression of genes related to carotenoid synthesis, and thus the relative content of carotenoids, is higher in the leaves of *U. pumila* cv. ‘Zhonghuajinye’ than in those of *U. pumila* [73]. This may partially explain the golden yellow leaf color of *U. pumila* cv. ‘Zhonghuajinye’. Under reduced light, the chloroplast structure of *U. pumila* cv. ‘Zhonghuajinye’ gradually returns to normal, which alters the expression of genes related to chlorophyll synthesis and metabolism, including *HemB*, *HemE*, *HemF*, and *HemY*. The relative content of chlorophyll therefore increases, causing greening of the golden yellow leaves [7,91].

Seasonal changes in the leaf color of *U. pumila* cv. ‘Zhonghuajinye’ have also been examined. The seasonal leaf color changes in *U. pumila* cv. ‘Zhonghuajinye’ are under the integrated regulation of metabolic pathways such as chlorophyll, carotenoids, and flavonoids. A study using weighted gene co-expression network analysis (WGCNA) identified a gene, *UpCrtR-b*, related to carotenoid synthesis. Its overexpression in tobacco significantly increased carotenoid accumulation, such that tobacco leaves turned yellow [72].

Leaf albinism has also been observed in *U. pumila* and *U. pumila* cv. ‘Zhonghuajinye’. In both, genes related to chlorophyll synthesis and photosynthesis are expressed at lower levels in white leaves. The low content of photosynthetic pigments and the resulting poor photosynthetic performance may be related to an abnormal chloroplast structure [27,71].

### 3.3. Biotic Stress

In natural environments, plant growth and development are often threatened by biological stresses in the form of infections by fungi, bacteria, viruses, and insects. In China, *Ulmus* is vulnerable to over 200 pest species. These have mainly been classified as drilling column pests, leaf-eating pests, and piercing sucking pests [92]. The main diseases of *Ulmus* are elm canker, elm anthracnose, black spot of elm, and Dutch elm disease (DED). Most of the pathogens are fungi, but in some cases, pathogenic organisms can be transmitted by pests [93,94]. DED, caused by pathogenic fungi of *Ophiostoma*, is one of the most destructive diseases of *Ulmus*. The two major outbreaks of DED, in Europe and North America, during the last century severely impacted local *Ulmus* populations [95]. Transcriptome analyses performed to investigate the pathogenic process of DED revealed that in DED-resistant *Ulmus*, the expression of genes such as *RPM1*, pathogenesis-related genes, phenylpropanoid biosynthetic pathway genes, and genes related to lignin polymerization was enhanced following infection. *Ulmus* may therefore employ a strategy of effector-triggered immunity to combat the invasion of pathogenic fungi [78]. In further research, a co-expression network comprising pathogen genes expressed during *Ulmus* infection was constructed, identifying a large number of candidate pathogenicity genes. Their further study will aid in elucidating the interaction mechanisms between *Ulmus* and pathogenic fungi [80].

In *Ulmus* leaves damaged by certain insects, abnormal tumors or protrusions, known as galls, may develop [96,97]. Related studies have shown that the jasmonic acid signaling pathway is mostly defective in gall tissues, suggesting the involvement of this pathway in their formation [83]. A large number of genes related to oxidative stress defense and signaling pathways may also be activated during gall formation [85]. The up-regulation of genes associated with cell proliferation and respiration during the initial stages of insect gall development was demonstrated in comparative transcriptome analyses conducted across various stages of insect gall formation. Among the genes markedly up-regulated during the insect gall formation and growth phases are those encoding lipoxygenases, glutathione S-transferases, superoxide dismutases, and protease inhibitors. During the insect gall opening phase, the expression of genes encoding lignocellulose synthesis enzymes is increased. These insights provide information to help elucidate the molecular regulatory mechanisms governing the development of insect galls [84].

### 3.4. Abiotic Stress

As plants are fixed organisms that cannot move freely in the natural environment, they are unable to avoid abiotic stresses, such as drought, high temperature, cold, and waterlogging, during their growth and development [98]. Anthropogenic abiotic stresses, such as air pollution, chemical pollution, microplastic pollution, and heavy metal pollution, also increasingly threaten plant growth [99,100,101]. However, through long-term natural selection, plants have evolved multiple mechanisms to cope with many abiotic stresses and maintain normal life activities [102]. *Ulmus* is highly resistant to abiotic stress, and the molecular mechanisms responsible for this resistance can be analyzed using transcriptomics techniques. The data obtained in such studies are important to support the breeding of stress-resistant trees.

High-salt environments have become common; their effects on plants include ionic stress, osmotic stress, and secondary damage. A transcriptome study of *U. pumila* under high salt stress showed the enrichment of genes in biological pathways such as photosynthesis, carbon fixation, and plant hormone signaling. The overexpression of *UpPETH* and *UpWAXY*, previously detected by WGCNA, can significantly improve the salt tolerance of *Arabidopsis thaliana* [65]. Further research has shown that certain genes involved in the regulation of circadian rhythm (such as *CRY2*, *ELF3*, *ZTL*, and *PRR5*) may also regulate the response of *U. pumila* to salt stress by affecting photosynthesis, thiamine metabolism, plant hormone signaling, and MAPK signaling pathways [64]. A study of *U. pumila* under high salt stress identified 303 miRNAs that responded to high salt stress. These miRNAs were shown to target and regulate 232 mRNAs, including those with a crucial role in the resistance of *U. pumila* to abiotic stress [68].

In their study on the response of *U. pumila* cv. ‘Zhonghuajinye’ to water stress, Zhang et al. revealed the differentially expressed genes are associated with biological pathways such as photosynthesis, starch and sucrose metabolism, tyrosine metabolism, the biosynthesis of abscisic acid, and amino sugar and nucleotide sugar metabolism. The expression of these genes promoted the accumulation of osmotic substances that enhanced the drought tolerance of *U. pumila* cv. ‘Zhonghuajinye’ [76]. Studies on *U. minor* demonstrated important roles for transcription factors such as MYB, DREB, HSF, and LEA proteins in the response to water stress [81]. A comparative transcriptome study of *U. wallichiana* during summer and winter revealed a complex and dynamic regulatory process in response to seasonal changes, including seasonal differences in the expression of *DREB* genes, which are thought to regulate plant tolerance to cold and drought stress [77].

## 4. *Ulmus* Metabolomics Research

The diverse edible components and medicinal properties of *Ulmus* have stimulated research into the nutritional and pharmacological properties of *Ulmus* [103,104,105,106,107]. Metabolomics, which enables qualitative and quantitative analyses of all small-molecule metabolites in biological tissues or cells during a specific period, can reflect the physiological and biochemical status of the organism [108].

Despite its potential for broad application in *Ulmus*, metabolomics research has been conducted only with respect to its medicinal components, biotic stress, and abiotic stress. The seeds and bark of *U. parvifolia* are rich in medicinal compounds and have been widely used in the treatment of inflammation. In a metabolomics study of the seeds and bark of *U. parvifolia* by Yin et al., 574 differentially expressed metabolites shared between the two organs were detected, including various bioactive compounds with antioxidant, anti-inflammatory, and anticancer activities, such as flavonoids, terpenosides, triterpenes, and sesquiterpenes. Seeds contained the highest contents of flavonoids and sesquiterpenes, while bark were mainly composed of terpenoid glycosides and triterpenoids [109].

Metabolomics was also used to analyze the physiological response of *Ulmus* to drought stress, with the results showing that *Ulmus* regulates cell osmotic pressure and prevents oxidative damage to their cells by increasing the cellular content of soluble sugars and amino acids. Specifically, under mild to moderate drought stress, the changes in primary metabolites were not significant, but the levels of raffinose and myo-inositol increased while those of citrate and malate decreased. During severe drought, there is a significant elevation in the contents of most amino acids as well as in the levels of mannitol, fructose, and glucose, among other metabolites [110,111].

Different species of *Ulmus* react inconsistently to the pathogen causing DED. When *Ophiostoma novo-ulmi* was inoculated onto *U. laevis*, *U. glabra*, and *U. minor*, the most severe response was that of *U. minor*, including a significant change in one-third of the metabolite content even 14 days post-inoculation. Under conditions of adequate irrigation followed by pathogen inoculation, metabolites such as isoleucine, phenylalanine, tryptophan, myo-inositol, and raffinose increased, whereas under conditions of drought followed by pathogen inoculation, metabolites such as GABA, glutamate, quinate, glucose, mannose, and sucrose decreased. The same study found that, 120 days after pathogen inoculation, *U. minor* exhibited the weakest symptoms of DED, indicating that the strong early changes in metabolites provided a degree of protection against the pathogen [110].

## 5. Current Problems and Prospects

### 5.1. Insufficient Assistance of Omics Data in the Study of Ulmus

Omics data have undeniably paved the way for advancements in the phylogenetic study of *Ulmus* species. However, unresolved issues persist, and nuclear genomic data are crucial for unlocking these mysteries. Despite the current research landscape, the absence of an officially published *Ulmus* species genome is a considerable hindrance to phylogenetic research. Fortunately, there is still hope, as full-length transcriptome sequencing or simplified genome sequencing offers feasible alternatives for obtaining detailed nuclear genome genetic information. It is important to note, however, that these advanced omics techniques have been applied to only a few species [42,43,112]. Nonetheless, given the valuable insights that can be obtained with these methods, whole-genome sequencing, full-length transcriptome sequencing, and simplified genome sequencing studies of *Ulmus* species should be supported and prioritized, as they will provide a foundation for the phylogenetic, resource conservation, and development and use of *Ulmus*.

Despite the large amount of published omics data on *Ulmus*, especially transcriptome sequencing and chloroplast genome data, they have not been fully used in gene mining and other applications. Most of the existing transcriptomes for *Ulmus* species are based on second-generation sequencing technology without a reference genome, such that the completeness of the gene structures and the accuracy of gene expression quantification need to be improved. Moreover, transcriptome sequencing has been limited to a few species, particularly *U. pumila* and *U. pumila* cv. ‘Zhonghuajinye’. In addition, several sets of chloroplast genome data have been made public for certain species of *Ulmus*. For example, published chloroplast genome data of *U. parvifolia* and *U. americana* cover six and five individuals, respectively. However, the majority of chloroplast genome data have primarily been used for phylogenetic research, with fewer applications in other areas, such as the development and application of SSR molecular markers and DNA barcoding or the analysis of chloroplast genome variation. Existing omics data thus remain to be further exploited as a driving force for scientific innovation.

### 5.2. Gene Mining and Functional Research of Ulmus

*Ulmus* has existed for over 65 million years. During their long evolution and through natural selection, *Ulmus* has evolved into many species with differing biological characteristics. For example, most *Ulmus* species bloom in spring, but *U. parvifolia* blooms in autumn. Meanwhile, most *Ulmus* species are deciduous, with the exception of *U. lanceifolia*, which is evergreen [2]. Furthermore, *Ulmus* species have a wide distribution and strong adaptability, and they produce edible fruits as well as compounds of medicinal value. Elucidating these biological characteristics has been facilitated by omics techniques; nonetheless, research focusing on the gene functions and regulatory mechanisms of *Ulmus* species has mostly been limited to gene cloning, gene expression, and functional validation through heterologous transformation. We, therefore, propose forthcoming research centers on exploiting omics data to pinpoint and delve into significant functional genes and intensively analyze the mechanisms regulating gene expression. The follow-up utilization of genetic transformation techniques to investigate gene functions would then be able to offer invaluable points of reference for the genetic improvement of elms.

### 5.3. Genetically Engineered Breeding System for Ulmus

Omics research can yield rich genetic information for forest research. Genetically engineered breeding is an important way to transform the products of this research into practical applications. Tissue culture plays a crucial role in genetic engineering, and relatively complete tissue culture systems have been established for some *Ulmus* species, such as *U. laevis*, *U. glabra*, *U. parvifolia*, and *U. pumila* [113,114,115]. In addition, somatic embryos have been successfully induced using *U. glabra* leaves [116]. However, challenges in the development of tissue culture systems for *Ulmus* species remain, such as the low rooting efficiency of some *Ulmus* species and the varying capacities for regeneration and rooting of different genotypes of *Ulmus* species [113,117]. A model species of *Ulmus* that can be easily cultivated and genetically transformed would promote the application of *Ulmus* species omics data.

Among the many *Ulmus* species, genetic transformation has been reported only for *U. americana* and *U. procera* [118,119]. The scarcity of reports on the genetic transformation of *Ulmus* species may be due to technical challenges. In recent years, a number of genetic transformation technologies with simple operation and high transformation rates have been developed, such as gene delivery mediated by nanoparticles [120], cut–dip–budding transformation [121], and regenerative activity dependence in planta injection delivery [122]. Their application in the genetic transformation of *Ulmus* species may lead to an efficient genetic transformation system suitable for *Ulmus* species.

## Figures and Tables

**Table 1:** Chloroplastic genome characteristics of *Ulmus* species.

Number	Species	Genome Size (bp)	Length of LSC (bp)	Length of IRs (bp)	Length of SSC (bp)	GC Content (%)	Accession Number
1	*Ulmus bergmanniana*	159,767	88,193	26,297	18,980	35.5	MT165921
2	*Ulmus canescens*	159,187	87,699	26,376	18,736	35.6	MT165922
3	*Ulmus castaneifolia*	159,700	88,016	26,361	18,962	35.5	MT165923
4	*Ulmus changii*	159,376	87,958	26,330	18,758	35.6	MT165924
5	*Ulmus chenmoui*	159,528	87,938	26,296	18,998	35.5	MT165925
6	*Ulmus davidiana*	159,645	88,249	26,297	18,802	35.5	MT165927
7	*Ulmus densa*	159,322	87,910	26,324	18,764	35.6	MT165928
8	*Ulmus gaussenii*	159,699	88,015	26,361	18,962	35.5	MT165930
9	*Ulmus glabra*	159,305	87,916	26,348	18,693	35.6	MT165931
10	*Ulmus glaucescens*	159,342	87,973	26,306	18,757	35.6	MT165932
11	*Ulmus laciniata*	159,711	88,118	26,296	19,001	35.5	MT165933
12	*Ulmus lamellosa*	159,722	88,244	26,297	18,884	35.5	MT165935
13	*Ulmus macrocarpa*	159,684	88,048	26,299	19,038	35.5	MT165937
14	*Ulmus microcarpus*	159,795	88,408	26,288	18,811	35.5	MT165938
15	*Ulmus minor*	159,304	87,915	26,348	18,693	35.6	MT165939
16	*Ulmus prunifolia*	159,712	88,028	26,361	18,962	35.5	MT165941
17	*Ulmus pumila*	159,685	88,267	26,288	18,842	35.5	MT165942
18	*Ulmus szechuanica*	159,588	88,035	26,296	18,961	35.5	MT165945
19	*Ulmus uyematsui*	159,693	88,116	26,296	18,985	35.5	MT165947
20	*Ulmus wallichiana*	159,422	87,993	26,368	18,693	35.6	MT165948
21	*Ulmus davidiana* var. *japonica*	159,411	88,508	26,017	18,868	35.6	KY244083
22	*Ulmus pumila* cv. ‘zhonghuajinye’	159,113	87,994	26,317	18,485	35.6	
23	*Ulmus pumila* cv. Tenue	159,375	87,937	26,332	18,774	35.6	MW544029
24	*Ulmus mianzhuensis*	159,425	87,584	26,546	18,749	35.6	OQ130025
25	*Ulmus parvifolia*	159,233	87,800	26,317	18,799	35.6	MT165940
26	*Ulmus lanceifolia*	158,742	87,170	26,404	18,764	35.6	MT165936
27	*Ulmus serotina*	159,270	87,762	26,413	18,682	35.6	MT165944
28	*Ulmus crassifolia*	159,338	87,839	26,413	18,673	35.6	MT165926
29	*Ulmus alata*	159,353	87,792	26,406	18,749	36.6	MT165919
30	*Ulmus elongata*	159,165	87,654	26,410	18,691	35.6	MT165929
31	*Ulmus thomasii*	159,457	87,886	26,413	18,745	35.5	MT165946
32	*Ulmus americana*	159,085	87,600	26,410	18,665	35.6	MT165920
33	*Ulmus laevis*	159,019	87,529	26,420	18,650	35.6	MT165934
34	*Ulmus rubra*	159,202	87,717	26,410	18,665	35.6	MT165943

Note: The table data are compiled from the literature and the NCBI database [16,17,18,19]. “GC content” refers to the Guanine–Cytosine ratio.

**Table 2:** Estimation of genome size in *Ulmus* species.

Number	Species	2C^y^ (pg)	1Cx^x^ (pg)	Number of Bases (Gb)	Subgenus
1	*U. alata*	2.998–3.142	1.499–1.571	1.466–1.536	Subg. *Oreoptelea*
2	*U. americana* 2x	3.088–3.196	1.544–1.598	1.510–1.563	Subg. *Oreoptelea*
3	*U. americana* ‘Jefferson’ 3x	4.652	1.551	1.517	Subg. *Oreoptelea*
4	*U. americana* 4x	6.007–6.572	1.501–1.643	1.469–1.607	Subg. *Oreoptelea*
5	*U. crassifolia*	3.106–3.223	1.553–1.612	1.519–1.576	Subg. *Oreoptelea*
6	*U. elongata*	3.000	1.500	1.467	Subg. *Oreoptelea*
7	*U. laevis*	2.975–3.032	1.488–1.516	1.455–1.483	Subg. *Oreoptelea*
8	*U. serotina*	3.091	1.546	1.511	Subg. *Oreoptelea*
9	*U. thomasii*	2.975–3.201	1.488–1.601	1.455–1.565	Subg. *Oreoptelea*
10	*U. castaneifolia*	3.838–3.969	1.919–1.985	1.877–1.941	Subg. *Ulmus*
11	*U. changii*	3.721–3.891	1.861–1.946	1.820–1.903	Subg. *Ulmus*
12	*U. chenmoui*	3.874–3.979	1.937–1.990	1.894–1.946	Subg. *Ulmus*
13	*U. davidiana* var. *davidiana*	3.734–3.908	1.867–1.954	1.826–1.911	Subg. *Ulmus*
14	*U. davidiana* var. *japonica*	3.633–3.781	1.817–1.891	1.777–1.849	Subg. *Ulmus*
15	*U. davidiana* var. *uncertain*	3.649	1.825	1.784	Subg. *Ulmus*
16	*U. glabra*	3.947–4.058	1.974–2.029	1.930–1.984	Subg. *Ulmus*
17	*U. glaucescens* var. *glaucescens*	3.674	1.837	1.797	Subg. *Ulmus*
18	*Ulmus harbinensis*	3.804	1.902	1.860	Subg. *Ulmus*
19	*U. laciniata* var. *laciniata*	3.759	1.880	1.838	Subg. *Ulmus*
20	*U. laciniata* var. *nikkoensis*	3.961	1.981	1.937	Subg. *Ulmus*
21	*U. lamellosa*	3.771–3.955	1.886–1.978	1.844–1.934	Subg. *Ulmus*
22	*U. macrocarpa* var. *macrocarpa*	3.987	1.994	1.950	Subg. *Ulmus*
23	*Ulmus microcarpa* 3x	5.678	1.839	1.851	Subg. *Ulmus*
24	*U. minor*	3.724–4.104	1.862–2.052	1.821–2.007	Subg. *Ulmus*
25	*U. parvifolia*	3.837–3.919	1.919–1.960	1.876–1.916	Subg. *Ulmus*
26	*U. prunifolia*	3.874	1.937	1.894	Subg. *Ulmus*
27	*Ulmus pseudopropinqua*	3.732	1.866	1.825	Subg. *Ulmus*
28	*U. pumila*	3.671–3.92	1.836–1.960	1.795–1.917	Subg. *Ulmus*
29	*U. rubra*	3.77–4.006	1.885–2.003	1.844–1.959	Subg. *Ulmus*
30	*U. szechuanica*	3.711–3.781	1.856–1.891	1.815–1.849	Subg. *Ulmus*
31	*U. uyematsui*	4.023	2.012	1.967	Subg. *Ulmus*
32	*U. wallichiana*	4.165	2.082	2.037	Subg. *Ulmus*
33	*Ulmus villosa*	2.175–2.277	1.088–1.139	1.064–1.113	Subg. *Indoptelea*

Note: 2C^y^ represents the measured DNA content of one nucleus, and 1Cx^x^ represents the DNA content of a haploid chromosome [39].

**Table 3:** Research content on DNA molecular markers of *Ulmus* species.

Number	Types	Content	Species
1	AFLP	Variety identification and genetic diversity analysis.	*U. Minor*, *U. glabra*, *U. americana*, *U. laevis*, *U. parvifolia*, *Ulmus carpinifolia*, *U. rubra*, *U. pumila*, *U. bergmanniana*, *U. szechuanica*, *U. minor,* etc. [44,45,46,47].
2	RAPD	Genetic diversity analysis, linkage mapping of disease resistance-related genes, kinship identification, and variety identification.	*U. pumila*, *U. parvifolia*, *U. glabra*, *Ulmus plotii*, *U. minor*, *U. laevis*, *U. americana,* etc. [48,49,50,51,52].
3	RFLP	Variety identification and pathogen identification.	*U. americana*, *U. rubra*, *U. parvifolia*, *Ulmus wilsoniana*, *U. pumila,* etc. [49,53].
4	ISSR	Identification of kinship relationships and analysis of genetic diversity.	*U. pumila*, *Ulmus propinqua*, *U. davidiana*, *U. laevis*, *U. macrocarpa*, *U. laciniata*, *U. parvifolia*, *U. davidiana* var. *japonica*, *U. pumila* cv. ‘zhonghuajinye’, etc. [54,55,56].
5	SRAP	Analysis of genetic diversity.	*U. Lamellosa,* etc. [57].
6	SSR	Leaf morphology association analysis, genetic diversity analysis, and conservation of endangered species.	*U. minor*, *U. glabra*, *U. laevis*, *U. wallichiana*, *U. gaussenii*, *U. pumila,* etc. [58,59,60,61].
7	SNP/Indel	Phylogenetic analysis, genetic diversity analysis, and environmental adaptability analysis.	*U. microcarpa*, *U. castaneifolia*, *U. davidiana*, *U. chenmoui*, *U. prunifolia*, *U. szechuanica*, *U. changii*, *U. castaneifolia*, *U. davidiana* var. *japonica*, *U. pumila*, *U. minor*, *U. lamellosa*, *U. macrocarpa*, *U. wallichiana*, *U. laciniata*, *U. uyematsui*, *U. rubra*, *U. glabra*, *U. villosa*, *U. laevis*, *U. thomasii*, *U. alata*, *U. crassifolia*, *U. serotina*, *U. elongata*, *Ulmus mexicana*, *U. americana*, *U. parvifolia,* etc. [42,43].

**Table 4:** Research content on the transcriptome of *Ulmus* species.

Number	Species	Content	Mode of Analysis
1	*U. pumila* [6]	Growth of branches.	Non-reference
2	*U. pumila* [62]	Fruit development and nutritional elements.	Non-reference
3	*U. pumila* [63]	Growth of branches.	Non-reference
4	*U. pumila* [64]	Response to salt stress.	Non-reference
5	*U. pumila* [65]	Response to salt stress.	Non-reference
6	*U. pumila* [66]	Development of seeds.	Non-reference
7	*U. pumila* [67]	Development of molecular markers.	Non-reference
8	*U. pumila* [68]	Response to salt stress.	Non-reference
9	*U. pumila* [69]	Aging of seeds.	Non-reference
10	*U. pumila* [70]	Physiological characteristics of plants with different ploidy.	Non-reference
11	*U. pumila* [71]	Photosynthetic characteristics of albino plants.	Non-reference
12	*U. pumila* cv. ‘zhonghuajinye’ [27]	The high temperature caused the leaves to turn white.	Reference
13	*U. pumila* cv. ‘zhonghuajinye’ [7]	Shading causes the leaves to regreen.	Non-reference
14	*U. pumila* cv. ‘zhonghuajinye’ [72]	Changes in leaf color.	Non-reference
15	*U. pumila* cv. ‘zhonghuajinye’ [73]	Growth inhibition and leaf color changes.	Non-reference
16	*U. pumila* cv. ‘zhonghuajinye’ [74]	Sunburn caused the leaves to turn white.	Non-reference
17	*U. pumila* cv. ‘zhonghuajinye’ [75]	Physiological characteristics of plants with different ploidy.	Non-reference
18	*U. pumila* cv. ‘zhonghuajinye’ [76]	Response to drought stress.	Reference
19	*U. wallichiana* [61]	Development of molecular markers.	Non-reference
20	*U. wallichiana* [77]	Seasonal senescence and abiotic stress responses.	Non-reference
21	*U. americana* [78]	The transcriptional regulation of plants resistant to DED and plants susceptible to DED.	Non-reference
22	*U. americana* [79]	Research on the development and adaptive evolution of transcript information.	Non-reference
23	*U. americana* [80]	Analysis and identification of DED pathogenic genes.	Non-reference
24	*U. minor* [81]	Response to drought and pathogen stress.	Non-reference
25	*U. minor* [82]	The impact of insect egg deposition on resistance to pests.	Non-reference
26	*U. davidiana* var. *japonica* [83]	Transcriptional regulation of gall formation.	Non-reference
27	Other elm trees [84]	Transcriptional regulation of gall formation.	Non-reference
28	Other elm trees [85]	Transcriptional regulation of gall formation.	Non-reference
